# Origin of *Oryza sativa* in China Inferred by Nucleotide Polymorphisms of Organelle DNA

**DOI:** 10.1371/journal.pone.0049546

**Published:** 2012-11-15

**Authors:** Xin Wei, Rongsheng Wang, Lirong Cao, Nannan Yuan, Juan Huang, Weihua Qiao, Wanxia Zhang, Hanlai Zeng, Qingwen Yang

**Affiliations:** 1 Institute of Crop Sciences, Chinese Academy of Agricultural Sciences, Beijing, China; 2 College of Plant Science and Technology, Huazhong Agricultural University, Wuhan, China; 3 Institute of Rice Research, Guangxi Academy of Agricultural Sciences, Nanning, China; Virginia Tech, United States of America

## Abstract

China is rich of germplasm resources of common wild rice (*Oryza rufipogon* Griff.) and Asian cultivated rice (*O. sativa* L.) which consists of two subspecies, indica and japonica. Previous studies have shown that China is one of the domestication centers of *O*. *sativa*. However, the geographic origin and the domestication times of *O*. *sativa* in China are still under debate. To settle these disputes, six chloroplast loci and four mitochondrial loci were selected to examine the relationships between 50 accessions of Asian cultivated rice and 119 accessions of common wild rice from China based on DNA sequence analysis in the present study. The results indicated that Southern China is the genetic diversity center of *O*. *rufipogon* and it might be the primary domestication region of *O*. *sativa*. Molecular dating suggested that the two subspecies had diverged 0.1 million years ago, much earlier than the beginning of rice domestication. Genetic differentiations and phylogeography analyses indicated that indica was domesticated from tropical *O*. *rufipogon* while japonica was domesticated from *O*. *rufipogon* which located in higher latitude. These results provided molecular evidences for the hypotheses of (i) Southern China is the origin center of *O*. *sativa* in China and (ii) the two subspecies of *O*. *sativa* were domesticated multiple times.

## Introduction

China is one of the most significant domestication centers. More than one hundred plants were domesticated by ancient Chinese people, such as *Setaria italica*, *Glycine max*, *Camellia sinensis*, *Oryza sativa*, etc [1]. *O*. *sativa*, also known as Asian cultivated rice, is the most important crop in China today. It takes about 30% of the cultivated land and feeds over 50% population of China. Owing to its great significance, the origin and domestication of *O*. *sativa* have been studied for decades. And previous studies have proved that *O*. *sativa* was domesticated from common wild rice (*O*. *rufipogon* Griff.) about 10000 years ago in China [2], [3]. However, several crucial questions about the domestication of *O*. *sativa* are still under debate.

One fundamental question still being argued is the geographic origin of *O*. *sativa* in China. Over centuries of evolution and domestication, the germplasm resources of both *O*. *sativa* and *O*. *rufipogon* are abundant with their extremely wide distribution in China. *O*. *sativa* is cultivated in more than twenty provinces while *O*. *rufipogon* grows in seven provinces: Fujian, Hunan, Jiangxi, Yunnan, Guangdong, Guangxi and Hainan, particularly with higher concentration in the last three provinces. According to the previous studies, three regions including Southern China, Yunnan-Guizhou highland and the middle and lower region of Yangtze River have been supposed to be the origin center of *O*. *sativa* based on evidences from the distribution of *O*. *rufipogon*, the rich germplasm resources of *O*. *sativa* and the discovery of rice phytoliths [4], [5], [6], [7]. However, the existing evidences supporting the mentioned hypotheses are far from being enough.

Another essential question is how many times that *O*. *sativa* has been domesticated. There are two subspecies of *O*. *sativa*, indica and japonica, which could be distinguished by a number of physiological and morphological traits such as drought tolerance, potassium chlorate resistance, phenol reaction, plant height, and leaf color, etc. There were two hypotheses about the domestication progress of the two subspecies: one has been popular for decades and is still argued in some papers recently, suggesting that indica and japonica were domesticated from one population of *O*. *rufipogon*, which was known as ‘Single Origin’ [8], [9], [10]; while the other has obtained much support from several genetic distance studies, suggesting that indica and japonica were domesticated separately from different *O*. *rufipogon* progenitors (namely ‘Multiple Origin’) [11], [12]. At present, whether indica and japonica were domesticated from a single or multiple domestication events is still being argued. A question closely related to this puzzle is when indica and japonica diverged. If indica and japonica were domesticated from the same group of *O*. *rufipogon*, the divergence might occur during the artificial selection. But if the divergence had completed before the domestication, the two subspecies must have been domesticated from two differentiated *O*. *rufipogon* groups.

In the northern hemisphere, the Tropic of Cancer (TOC, 23.5° N) represents the northernmost position where the sun is directly overhead at the June solstice, and is the recognized boundary for tropical and subtropical rice. *O*. *rufipogon* in China could be divided into two groups by the TOC, tropical *O*. *rufipogon* and subtropical *O*. *rufipogon*. One study based on the photoperiod genes had suggested that both indica and japonica had closer relationship with tropical *O*. *rufipogon* than subtropical *O*. *rufipogon* [13]. Whether the two subspecies show close affinity to tropical *O*. *rufipogon* or subtropical *O. rufipogon* in organelle genomes would be examined in the present study. Since *O. sativa* was more likely to be domesticated from the *O. rufipogon* group which had closer relationship with it, whether tropical *O. rufipogon* or subtropical *O. rufipogon* was the ancestor of *O. sativa* also could be revealed by the examination.

The DNA of organelles has been widely used in the phylogenetic analysis because of its slower nucleotide substitutions rates, uniparental inheritance and absence of intermolecular recombination [14], [15]. Ten fragments from chloroplast and mitochondrial genomes were chosen and sequenced to determine the origin and domestication process of *O*. *sativa* in China. Among the ten fragments, six loci were from chloroplast genome and four loci were from mitochondrial genome. And intergenic spaces, introns and coding regions all were included in.

In the present study, we would like to answer the following questions:

1. What is the diversity of the organelle genes in *O*. *sativa* and *O*. *rufipogon*?
2. Whether indica and japonica were domesticated from one group or multiple groups of *O*. *rufipogon* and when did they diverge?
3. Where was the domestication center of *O*. *sativa* in China?
4. Were indica and japonica domesticated from tropical *O*. *rufipogon* or subtropical *O*. *rufipogon*?

## Materials and Methods

### Sampling and Choice of Loci

The materials used in this study included 50 accessions of cultivated rice, one accession of *O*. *barthii* and 119 accessions of *O*. *rufipogon* (Table S1). Distributions of the samples were shown in Figure S1. The cultivated accessions are all landrace (pure-line varieties developed by farmers without artificial intercrossing), containing 27 indica and 23 japonica cultivars from 25 provinces in China. The subtropical *O*. *rufipogon* group included 46 accessions from Fujian, Guangdong, Guangxi, Hunan, Yunnan and Jiangxi provinces, while the tropical *O*. *rufipogon* group consisted of 73 accessions from Guangdong, Guangxi, Hainan and Yunnan provinces. The *O*. *rufipogon* accessions had been investigated carefully in the whole life in case that the individuals with gene flow from *O. sativa* were not included in the samples. All *O*. *sativa* and *O*. *rufipogon* were chosen from the Chinese rice core collection with high diversity. The mini-core collections of *O*. *sativa* and *O*. *rufipogon* in China were selected from the Chinese national germplasm collections which included 50526 landraces and more than 10000 accessions of *O*. *rufipogon*. Through a hierarchical sampling strategy, mini-core collections of *O*. *sativa* and *O*. *rufipogon* retained more than 70% of the morphological variation in all germplasm collections [16]. Typical indica and japonica varieties from the mini-core collections of *O*. *sativa* and also most all individuals of mini-core collections of *O*. *rufipogon* have been selected and used in the present study. *O*. *sativa* were obtained from Chinese National Germplasm Bank and *O*. *rufipogon* was procured from Guangzhou and Nanning Wild Rice Germplasm Banks. *O*. *barthii* was provided by the International Rice Research Institute and used as outgroup. To distinguish indica and japonica, we had investigated all individuals by Cheng’s Index Method [17] which has been popularly used in China to identify indica and japonica.

Six fragments of chloroplast genome (*trnG-trnfM*, *atp1*, *trnT-trnL*, *trnC-ycf6*, *nhdC-trnV*, and intron of *rps16*) and four fragments of mitochondrial genome (*cox3*, *cox1*, *rps2-trnfM*, and intron of *nhd4*) were selected and sequenced for all samples (Table S2). Among these loci, *trnG-trnfM*, *trnT-trnL*, *trnC-ycf6*, *nhdC-trnV* and *rps2-trnfM* were the internal sequences; *rps16* and *nhd4* were introns; *atp1*, *cox3* and *cox1* were the coding regions.

### DNA Extraction, PCR Amplification, and Sequencing

Chloroplast DNA and mitochondrial DNA were extracted from fresh seedling leaves [18] and nuclear DNA had been cleared out completely. All of the amplifications with polymerase chain reaction (PCR) were performed in a total of 25 µl reaction mixture using a TProfessional Thermocycler (Biometra, Germany) with 10–30 ng genomic DNA. The reaction mixture included 0.2 µM of each primer, 200 µM of each dNTP, 10 mM Tris-HCl (pH = 8.3), 50 mM KCl, 1.5 mM MgCl_2_, and 0.5 U HiFi DNA polymerase (Transgen, China). The amplification conditions were 94°C for 5 min followed by 35 cycles of 94°C (30 s), 55°C (30 s), and 72°C (1.5 min), and a final extension at 72°C (10 min). The PCR products were electrophoresed in 1.2% agarose gels, and the DNA fragments were cut from the gel and purified using the Tiangen Gel Extraction kit (Tiangen, China). Sequencing reactions were performed by an ABI 3730 automated sequencer (Applied Biosystems, United States). Because Taq errors did occur, when polymorphisms were only found in one of the accessions, this accession was re-sequenced with the cloning step to ensure those results were not false polymorphisms.

### Statistical Analysis

The DNA sequences were aligned using the ClustalX 1.83 program [19] and manually adjusted in BioEdit [20]. Insertions/deletions (indels) were not included in the analysis. We calculated the number of segregating sites (S), the number of haplotypes (h), haplotype diversity (Hd) and two parameters of nucleotide diversity, (π) [21] and Watterson’s estimator from S (θ_w_) [22], using DNAsp version 5.0 [23]. Pairwise F_ST_, generally expressed as the proportion of genetic diversity due to allele frequency differences among populations, was used to measure differentiation between groups, as implemented in Arlequin 3.01 [24].

Haplotype network was constructed by mutational steps with NETWORK 4.5 [25]. Those networks represent the genetic distance of DNA sequences or alleles and were mainly composed of circles of different sizes and colors and lines that linked those circles. The circle size is proportional to the number of samples within a given haplotype, and the lines between the haplotypes represent mutational steps between the alleles. The numbers next to the circle represent the haplotype number. Each color of the circles represents a species or subspecies. If more than one nucleotide difference existed between the linked haplotypes, it is indicated by a number next to the lines.

The phylogenetic relationships among the haplotypes of the three nuclear loci and one combined chloroplast and mitochondrial gene region were constructed by Neighbor-joining (NJ) [26] analysis using PAUP* version 4.0b10 [27]. Gaps were treated as missing values, and these sites were excluded from the data matrix. In the NJ analysis, we chose to follow Kimura’s 2-parameter (K2P) model [28] and the nonparametric bootstrap test was performed to quantify the confidence level of internal nodes with 1000 replications.

Structure analysis was used to assign individual clusters to groups for all genes. A Bayesian approach assigned individuals to a specific number of clusters (K) based on inferred allele frequencies in populations [29]. The optimal number of genetic clusters was identified using log likelihood, based on independent runs at K = 2–10. All runs were iterated for 100 000 Markov chain Monte Carlo sampling steps following 100 000 burn in steps and a thinning interval of 10 steps. Each individual consisted of a mixture of different genomic components referred to by different colors. If the color of one cluster was more than 60% in one individual, we supposed it belonged to this cluster.

Because the substitution rates of the plant mitochondrial, chloroplast, and nuclear DNA had been reported to approximate at 1∶3∶12 [30] and the evolutionary rate of rice nuclear genes had been estimated to be 7.16 × 10^−9^ [31], we estimated the divergence time between indica and japonica by a molecular clock, using the formula T = 3N/Lµ for chloroplast genes and T = 12N/Lµ for mitochondrial genes, where µ corresponds to the absolute rate of substitutions per site per year of nuclear genes, N is the estimated numbers of substituted sites between indica and japonica, and L is the total length of all loci.

## Results

### Nucleotide Diversity

We sequenced ten unlinked organelle loci included *rps133*, *trnG-trnfM*, *atp1*, *trnT-trnL*, *trnC-ycf6*, *nhdC-trnV*, *cox3*, *cox1*, *nhd4* and *rps2-trnfM* for 50 accessions of *O*. *sativa* and 119 accessions of *O*. *rufipogon*. The total length of the ten loci was 9089–9233. The number of insertion-deletion (indel) events ranged from 7 to12.

Standard statistics of sequence polymorphisms for all loci are shown in Table 1. For *O*. *sativa*, 11 and 3 polymorphisms were found in chloroplast and mitochondrial, respectively. For *O*. *rufipogon*, more polymorphisms were identified, including 15 and 8 polymorphisms in chloroplast and mitochondrial, respectively. θ_w_, which represents the diversity of the nucleotide polymorphisms of *O*. *sativa*, was 0.45 and 0.18 in chloroplast and mitochondrial, respectively. θ_w_ of *O*. *rufipogon* also was higher than that of *O*. *sativa*, being 0.52 and 0.41 in chloroplast and mitochondrial, respectively. More polymorphisms and higher diversity of polymorphisms indicated that genetic diversity of the *O*. *rufipogon* was higher than that of *O*. *sativa* in chloroplast and mitochondrial genes. As expected, the values of S, h, Hd, π and θ_w_ of chloroplast loci all were higher than mitochondrial loci. This result was in line with the fact that the mitochondrial DNA is more conservative and the evolution rate of chloroplast DNA is higher than mitochondrial DNA in rice.

**Table 1:** Summary of nucleotide polymorphisms.

Organelle	Species	S	h	Hd	π×10^3^	θ_w_×10^3^
Chloroplast	*O*. *sativa*	11	3	0.548	0.95	0.45
	*O*. *rufipogon*	15	10	0.771	1.13	0.52
Mitochondrial	*O*. *sativa*	3	2	0.507	0.42	0.18
	*O*. *rufipogon*	8	7	0.554	0.44	0.41
Combined	*O*. *sativa*	14	3	0.548	0.74	0.34
	*O*. *rufipogon*	23	15	0.793	0.85	0.47

S: number of segregating sites; h: number of haplotypes; Hd: haplotype diversity; π: nucleotide diversity; θ_w_: Watterson’s parameter for silent sites.

### Haplotype Variation

**Figure 1:**
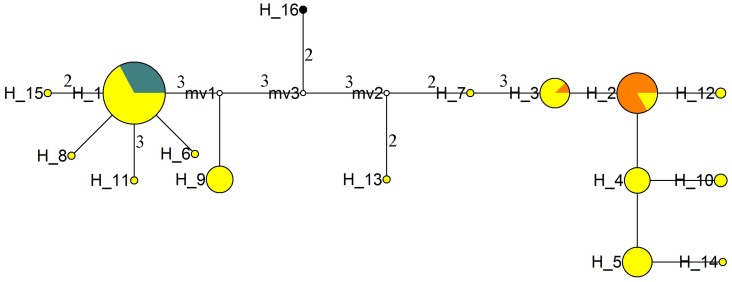
Haplotype network constructed by all loci for *O*. ***sativa*** **and** ***O***. ***rufipogon*** **.** The circle size is proportional to the quantity of samples within a given haplotype, and the numbers next to the circle represent the haplotype number. Lines between haplotypes represent mutational steps between alleles. When more than one nucleotide difference existed between linked haplotypes, this is indicated by the numbers next to the lines. Colors for species: yellow, *O*. *rufipogon*; orange, *O*. *sativa indica*; blue, *O*. *sativa japonica*; and black, *O*. *barthii*.

One haplotype of *O*. *barthii*, three haplotypes of *O*. *sativa* and fifteen haplotypes of *O*. *rufipogon* were found for all loci. Figure 1 shows the network constructed by all haplotypes. All *O*. *sativa* were included in H1, H2 and H3, and a great many of *O*. *rufipogon* also existed in these haplotypes and shared the haplotypes with *O*. *sativa*. Since *O*. *sativa* was most likely domesticated from the *O*. *rufipogon* individuals with the same nucleotide variations, it could be concluded that the wild accessions in H1, H2 and H3 were the ancestors of the cultivated accessions. Furthermore, in this haplotype network, japonica accessions were only included in H1 and indica accessions were contained in H2 and H3. Thus, we concluded that japonica was domesticated from the *O*. *rufipogon* in H1 and indica was domesticated from the *O*. *rufipogon* in H2 and H3.

In the network, H16, which represented the outgroup from *O*. *barthii*, was in the middle and divided the other haplotypes into two groups. One group included H1, H6, H8, H9, H11, H15 and the other group contained H2, H3, H4, H5, H7, H10, H12, H13 and H14. This result suggested that *O*. *rufipogon* might have already diverged into two groups. As analyzed above, H1 and H2, H3 were the direct progenitors of japonica and indica respectively. These two groups could be named as indica-like and japonica-like groups.

### Phylogenetic Analysis

**Figure 2:**
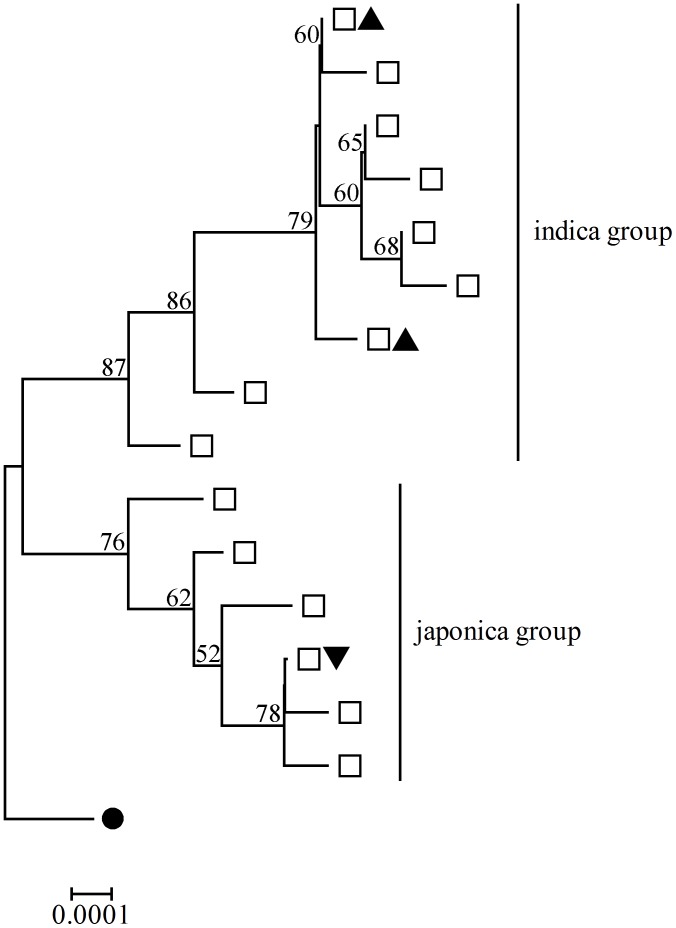
Phylogenetic tree of combined chloroplastic and mitochondrial loci. Bootstrap values above 50% are shown on the trees. The accessions contained in the branches were indicated by different symbols: *O*. *rufipogon* alleles by open squares, indica alleles by filled triangles and japonica by inverted filled triangles. The tree was rooted with *O*. *barthii* allele which is indicated by solid circles.

The phylogenies tree of the combined chloroplast and mitochondrial loci were constructed by NJ method (Figure 2). Because of the overall similarity between *O*. *sativa* and *O*. *rufipogon*, the phylogenetic tree should not be treated as true genealogies but rather an approximation of genealogy [32]. As shown in Figure 2, all branches were clearly divided into two groups, and the branches shared by indica and japonica with *O*. *rufipogon* were in the upper and lower groups respectively. The phylogenetic analysis strongly supported that japonica and indica were domesticated from japonica-like *O*. *rufipogon* and indica-like *O*. *rufipogon* independently.

### Structure Analysis

**Figure 3:**
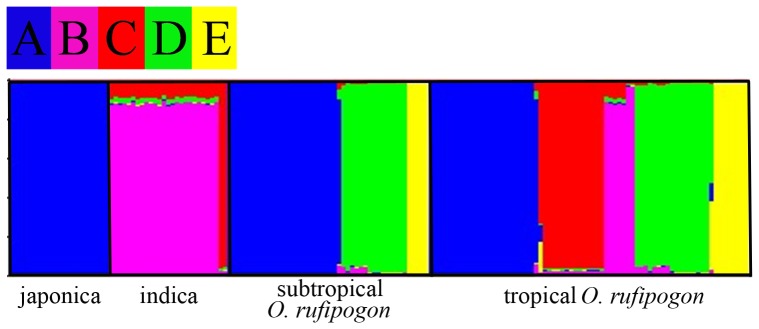
Population structuring of *O*. ***sativa*** **and** ***O***. ***rufipogon*** **.** STRUCTURE was constructed by all loci. K = 5. Clusters are indicated by different colors. Samples included in all clusters are listed in Table S1.

Structure analysis for all *O*. *sativa* and *O*. *rufipogon* was performed from K = 2 to K = 10. When K = 5, the value of Ln P (D) was the largest (Figure S2) and the result was stable. Five clusters were identified for all samples (Figure 3), among which three were shared by both *O*. *sativa* and *O*. *rufipogon*. Indica and japonica was clearly separated, and japonica fell into cluster A and indica was divided into cluster B and C. Cluster A, B and C were also included in *O*. *rufipogon*. *O*. *rufipogon* in the same clusters of indica and japonica might be the progenitors of indica and japonica respectively. This result also supports the conclusion that japonica and indica were domesticated from different *O*. *rufipogon* groups.

What’s more, cluster B and C were only detected in tropical *O*. *rufipogon*, revealing indica was domesticated from tropical *O*. *rufipogon* rather than subtropical *O*. *rufipogon*. While cluster A were contained both in tropical *O*. *rufipogon* and subtropical *O*. *rufipogon*, indicating a more wide geographic origin of japonica.

### Molecular Dating of the Divergence of Indica and Japonica

In total, twenty three SNPs had been detected in chloroplast and mitochondrial loci for all *O*. *sativa* and *O*. *rufipogon*. Among these SNPs, some could be used to distinguish indica and japonica varieties and divide the *O*. *rufipogon* into indica-like and japonica like groups. Ten and three SNPs of this kind had been found in chloroplast loci and mitochondrial loci respectively. Using the formulas T = 3N/Lµ for chloroplast loci and T = 12N/Lµ for mitochondrial loci, we estimated the divergence time for indica and japonica were 0.08 million years ago (mya) in chloroplast genome and 0.13 mya in mitochondrial genome. Thus, we concluded that the divergence of indica and japonica were completed at about 0.1 mya.

### Distribution of the Ancestors of *O*. *sativa*

**Figure 4:**
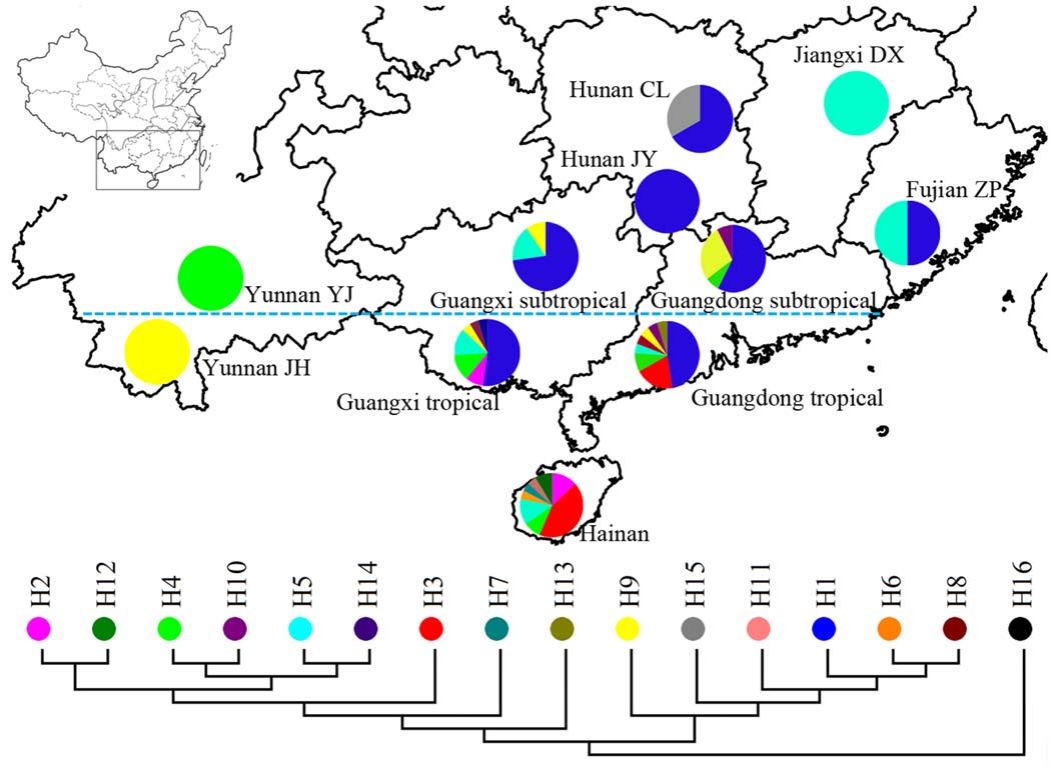
A map showing the sampled populations of *O*. ***rufipogon*** **and the distribution of haplotypes.** Detailed information of the samples is provided in Table S1. Phylogenetic relationship of the haplotype based on the NJ analysis is indicated below the map. Pie charts show the proportions of the haplotypes within each population. Haplotypes are indicated by different colors. The Tropical of Cancer is indicated by the green dotted line. Codes: CL, Chaling; DX, Dongxiang; JH, Jinghong; JY, Jiangyong; YJ, Yuanjiang; ZP, Zhangpu;

**Figure 5:**
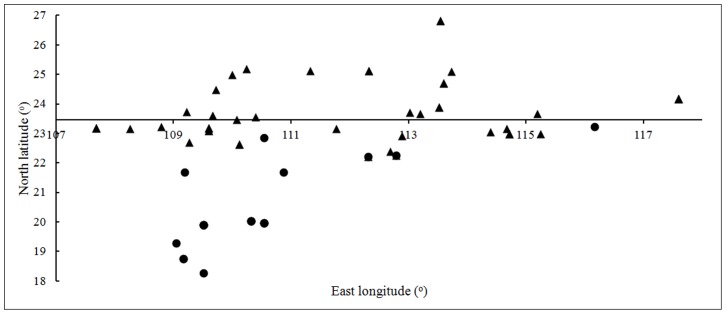
Longitude and latitude of the *O*. ***rufipogon*** **from H1, H2 and H3.** Accessions from H2 and H3 are indicated by circles and accessions from H1 are indicated by triangles. The horizontal ordinate origins from 23.5° (N) which is the Tropical of Cancer located in.

The distribution of fifteen haplotypes of *O*. *rufipogon* was shown in Figure 4. And we found that the haplotypes were closely related to particular geographic locations. H1 existed in Guangdong, Guangxi, Fujian and Hunan; H2 existed in Guangxi tropical and Hainan; H3 were included in Guangdong tropical and Hainan. Because *O*. *rufipogon* contained in H1 might be the ancestors of japonica are *O*. *rufipogon* included in H2 and H3 might be the ancestors of indica, we supposed ancestors of japonica mainly grew in Guangdong, Guangxi, Fujian and Hunan while ancestors of indica mainly existed in Guangdong tropical, Guangxi tropical and Hainan. To find out the exact origin of indica and japonica, a coordinate diagram had been constructed by the longitude and latitude of the *O*. *rufipogon* accessions in H1, H2 and H3 (Figure 5). Locations of the individuals in H1 ranged from 22°15′ (N) to 26°48′ (N) while locations of the accessions in H2 and H3 ranged from 18°15′ (N) to 23°18′ (N). The latitude of the distribution of ancestors of indica was lower than that of japonica, indicating that indica and japonica might be domesticated from different regions in Southern China. This map also showed that ancestors of indica located in south of the TOC and ancestors of japonica mainly located in north of the TOC and there were only a few accessions located in the south nearby the TOC. This result indicated indica might be domesticated from tropical *O*. *rufipogon* and japonica was domesticated from *O*. *rufipogon* which located in higher latitude region.

### Genetic Differentiation between *O*. *sativa* and *O*. *rufipogon*

Pairwise F_ST_ values were calculated to measure genetic differentiation between different *O*. *sativa* and *O*. *rufipogon* groups. The genetic differentiation between japonica and subtropical *O*. *rufipogon* was smaller than that between japonica and tropical *O*. *rufipogon*. In contrast, the genetic differentiation between indica and subtropical *O*. *rufipogon* was larger than that between indica and tropical *O*. *rufipogon* (Table 2). Assuming the different F_ST_ values are an indicator of genetic affinity, we proposed that indica had a closer relationship with tropical *O*. *rufipogon* while japonica had a closer relationship with subtropical *O*. *rufipogon*.

**Table 2:** Summary statistic of pairwise divergence (F_ST_) between groups.

Groups	Tropical *O*. *rufipogon*	Subtropical *O*. *rufipogon*	Tropical ancestors	Subtropical ancestors
japonica	0.5655	0.3526	0.4314	0
indica	0.2775	0.5089	0.4507	0.9962

## Discussion

### Genetic Diversity of *O*. *sativa* and *O*. *rufipogon* in China

The genetic diversity of nuclear genes in *O*. *sativa* and *O*. *rufipogon* has been investigated based on molecular markers, SNPs and indels [33], [34], [35], [36], [37]. These researches had suggested that there was a bottleneck in the domestication of *O*. *sativa*, and *O*. *rufipogon* from Southern China has the highest diversity. We also got the similar result in organelle DNA of *O*. *sativa* and *O*. *rufipogon* in China.

Although *O*. *sativa* accessions used in the present research were collected from the Chinese rice core collection and all are landrace, only three haplotypes were detected in all loci. Using the haplotype numbers as a proxy for diversity, *O*. *rufipogon* contained 100% of the total haplotype diversity, whereas *O*. *sativa* only contained about 20% of the total haplotype diversity of chloroplast and mitochondrial genome. The comparison of the levels of diversity between *O*. *sativa* and *O*. *rufipogon* indicated that *O*. *sativa* reduced an 80% subset of the total genetic variation of *O*. *rufipogon* in organelle genomes. The genetic diversity maintained in cultivated rice was much less than the wild progenitors, indicating a severe genetic bottleneck during domestication.

In the present study, haplotype numbers of *O*. *rufipogon* from each province were calculated (Figure S3). The haplotype numbers of *O*. *rufipogon* for all loci from Fujian, Guangdong, Guangxi, Hainan, Hunan, Jiangxi and Yunnan was 2, 8, 7, 8, 2, 1 and 2, respectively. This result indicated that *O*. *rufipogon* from Guangdong, Guangxi and Hainan (together being named as Southern China) had the highest organelle DNA diversity. The haplotype numbers of organelle DNA of *O*. *rufipogon* from Southern China was 14, taking up to 93% of the total haplotype numbers and about three times of that from all other provinces. Although this phenomenon might be caused by different numbers of sampled populations and the fact that common wild rice populations in the wild in other provinces are less than in Guangdong, Guangxi and Hainan, our results support the opinion that *O*. *rufipogon* from Southern China contained the most diversity of organelle DNA and Southern China might be the genetic diversity center of *O*. *rufipogon* in China.

There were 14 haplotypes of tropical *O*. *rufipogon* for all loci, containing most diversity of all *O*. *rufipogon*, while haplotype number of subtropical O. *rufipogon* was 6, only representing 40% of the total diversity. The genetic diversity of tropical *O*. *rufipogon* was much higher than subtropical *O*. *rufipogon*. This result may provide an explanation to the fact that the diversity of indica was higher than japonica because indica was domesticated from tropical *O*. *rufipogon* which contained higher genetic diversity.

### Domestication Model of *O*. *sativa* in China

Although the point that indica and japonica were domesticated independently has obtained much support [38], [39], [40], some papers published even recently still insisted that *O*. *sativa* was only domesticated once from group of *O*. *rufipogon* [8], [9], [10], [41], [42]. The opinions that whether indica and japonica were domesticated single or multiple times are considerably controversial [43], [44], [45], [46]. Our results strongly support the multiple origin model rather than the single one. In the present study, the phylogenetic analysis indicated that *O*. *rufipogon* diverged into two groups, indica-like and japonica-like, and plenty of individuals with the same polymorphisms of indica and japonica existed in indica-like and japonica-like *O*. *rufipogon*. It could be concluded that indica and japonica were domesticated separately because they had evolved from different ancestors. Since all *O*. *rufipogon* samples were selected from Chinese rice core collection which can highly represent the diversity of common wild rice in China and we had carefully monitored the whole life of the wild samples to ensure that the individuals with gene flow from *O*. *sativa* were not included in, we confirmed that those *O*. *rufipogon* accessions with the same polymorphisms of *O*. *sativa* did not inherit the organelle genomes from cultivated parent and were the direct progenitors of indica and japonica.

Recently, it's reported that the gene flow of domestication genes in nuclear genome from japonica to indica occurred during the domestication of *O*. *sativa* [39], [40]. However, this phenomenon was not detected in organelle DNA in this study. These results different from the previous researches in nuclear genome may have been caused by the uniparental inheritance of organelle genomes. Because the organelle genomes of the next generation are only inherited from the female parent, the introgression between indica and japonica rarely occurred.

It is believed that *O*. *sativa* was domesticated about 10000 years ago in East Asia [3]. The molecular dating in the present study revealed that indica and japonica diverged in about 0.1 mya, which indicated that the divergence time of indica and japonica was much earlier than the beginning of rice domestication. This result proved that indica and japonica had already separated in *O*. *rufipogon* before domestication. Divergence time of indica and japonica also has been calculated in previous researches [36], [47]. By analyzing the divergence of indica and japonica in nuclear genes, the two subspecies was supposed to separate approximately 0.4 mya. And based on the total number of nucleotide substitutions between the chloroplast genomes of 93-11 and PA64S, the divergence of indica and japonica was dated as 0.86 to 2 mya. Both studies of nuclear and chloroplast genes have revealed that the divergence of indica and japonica occurred much earlier than the beginning of rice domestication.

The genetic differentiation between tropical *O*. *rufipogon* and indica was significantly smaller than that between subtropical *O*. *rufipogon* and indica, suggesting indica has a closer relationship with tropical *O*. *rufipogon*. Furthermore, latitudes of the ancestors of indica in China shown in Figure 5 were all in the south of the TOC, from which we concluded that indica was domesticated from tropical *O*. *rufipogon*. Although the genetic differentiation analysis indicated japonica was closer to subtropical *O*. *rufipogon* than to tropical *O*. *rufipogon*, the distribution of the ancestors of japonica was not only in subtropical area but also in tropical region nearby the TOC. Thus japonica may have been domesticated from subtropical *O*. *rufipogon* or tropical *O*. *rufipogon* nearby the TOC.

Indica and japonica grow in different areas in China and adapt to different environments. Generally, indica grows in the lower latitude regions and adapts to a higher temperature and shorter light period, while japonica grows in higher latitude regions and adapts to a lower temperature and longer light period. The similar phenomenon was detected in the ancestors of indica and japonica. The latitude of the distribution of ancestors of indica was lower than that of the ancestors of japonica, suggesting ancestors of indica grew in the conditions of warmer climate and shorter light period. We supposed that the adaptability of warmer climate and shorter light period of the indica-like *O*. *rufipogon* had been inherited by indica, leading to the lower location of indica compared with japonica.

Domestication of rice in China also has been analyzed by nuclear genes such as *ITS*, *SS*, *Hd1*, *Ehd1* and *Waxy* by our group [42]. The results inferred by nuclear genes and organelle genes were not all the same. For neutral nuclear genes, such as *ITS* and *SS*, the revealed domestication process was quite similar to organelle genes. Indica and japona might have been domesticated from indica-like and japonica-like *O*. *rufipogon* groups. But for the domesticated gene such as *Hd1* and *Waxy*, the results were different. Functional *Hd1* gene in *O*. *sativa* evolved like orangelle genes, but nonfunctional *Hd1* gene might evolve from nonfunctional *Hd1* gene in *O*. *rufipogon*. For *Waxy*, it was first domesticated in japonica and then transferred into indica later. The domestication of nuclear genes is much more complex than that of organelle genes.

### Domestication Center of *O*. *sativa* in China

Six major haplotypes of *O*. *rufipogon* of organelle DNA were detected: H1, H2, H3, H4, H5 and H9. As shown in Figure 4, each haplotype was located in a limited area, and Guangdong and Guangxi were the center of the distribution region: H1 was included in the east and north direction of the center (Fujian, Guangdong, Guangxi and Hunan); H2 was included in south direction of the center (Guangxi and Hainan); H3 was also included in south direction of the center (Guangdong and Hainan); H4 was included in south and west direction of the center (Guangdong, Guangxi, Hainan and Yunnan); H5 was included in south and east direction of the center (Guangdong, Guangxi, Hainan, Fujian and Jiangxi); H9 was included in west direction of the center (Guangdong, Guangxi and Yunnan), suggesting Guangdong and Guangxi might be the genetic center of *O*. *rufipogon* in China and *O*. *rufipogon* in other areas may be derived from those of Guangdong and Guangxi.

The debate of the geographic origin of *O*. *sativa* in China is mainly focused on three regions with different evidences: Southern China, Yunnan-Guizhou Highland and the middle and lower region of Yangtze River. Southern China has been supposed to be the origin center of *O*. *sativa* because the ancestors of cultivated rice (*O*. *rufipogon*) only located in the eight provinces of South China [4]. Whereas, due to the highest genetic diversity of cultivated rice in Yunnan and Guizhou provinces, a hypothesis popular in the 1970s identified Yunnan-Guizhou highland as the origin site of *O*. *sativa* in Asia [5]. From 1970s, many rice phytoliths with long history were found in different archaeological sites in the middle and lower region of Yangtze River, some scientists deduced that this region was the geographic origin of rice domestication and cultivation in China [6], [7]. Since Guangdong and Guangxi belong to Southern China, Yunnan belongs to Yunnan-Guizhou Highland, Dongxiang county in Jiangxi belongs to the middle and lower region of Yangtze River, and *O*. *rufipogon* existed in all these regions, examining the relationship between *O*. *sativa* and *O*. *rufipogon* from these regions would provide molecular evidence to verify these hypotheses.

By analyzing SNPs of *O*. *rufipogon* accessions in different haplotypes, we found that *O*. *rufipogon* accessions in H1 had the same nucleotide polymorphisms with japonica and *O*. *rufipogon* accessions in H2 and H3 had the same nucleotide polymorphisms with indica, indicating *O*. *rufipogon* accessions in H1 and H2, H3 may be the ancestors of japonica and indica respectively. As we mentioned above, *O*. *rufipogon* individuals in H1 were from Fujian, Guangdong, Guangxi and Hunan while *O*. *rufipogon* individuals in H2 and H3 were from Guangdong, Guangxi and Hainan. This result clearly showed that *O*. *rufipogon* from Yunnan and Jiangxi were not the progenitors of *O*. *sativa* and *O*. *sativa* was domesticated from Southern China rather than from Yunnan-Guizhou Highland and the middle and lower region of Yangtze River.

Although the samples in Yunnan and Jiangxi are limited, they could highly represent the diversity in these areas. Thus, because *O*. *rufipogon* from Yunnan and Jiangxi are not in H1, H2 and H3, it could be concluded that *O*. *sativa* was not domesticated from these areas. In the history, the distribution of *O*. *rufipogon* might different from today, and *O*. *rufipogon* might be more than today in Yunnan and Jiangxi. The results in this study were concluded from the distribution of *O*. *rufipogon* currently in China. But we believed our analysis had explained the results quite well and these results could be helpful for understanding the domestication process of *O*. *sativa* in China. Previous phylogeographic study has suggested indica was domesticated in Southeast and South Asia whereas japonica originated from Southern China [12]. Another research published recently argued only indica was domesticated from China [10]. However, in the present study, both ancestors of indica and japonica were detected in Southern China. We supposed that not only japonica but also indica was domesticated in Southern China. To confirm whether indica was domesticated from Southeast and South Asia or only from Southern China, plenty representative *O*. *rufipogon* individuals from Southeast and South Asia and Southern China should be together included in the samples for further research. The question whether indica was domesticated from China or South Asia remains open.

Although the wild samples might have gene flow from cultivated rice, the results showed that plenty of wild samples had the same polymorphisms with indica and japonica respectively. These wild samples were in a wide range. It is not possible all these wild samples had gene flow from *O*. *sativa*. The introgression between *O*. *sativa* to *O*. *rufipogon* had been detected in our previous study [42] and the result indicated the introgression was at a low level. What’s more, the *O*. *rufipogon* accessions had been investigated carefully in the whole life to prevent that the individuals which had gene flow from *O*. *sativa* were not included in the samples. Therefore we thought the introgression from *O*. *sativa* to *O*. *rufipogon* is at a low level and human activities could rarely impact the conclusions of the study.

A recently published paper suggests O. rufipogon had two subpopulations: ruf I and ruf II and indica was domesticated from ruf I in China [10]. In the present study, the result clearly showed both japonca and indica were domesticated from *O*. *rufipogon*. The different conclusion in the mentioned paper might be caused by the limited samples from China. Only 22 accessions of *O*. *rufipogon* accessions from China had been used and the detailed information about these accessions was not provided in the paper. We supposed the *O*. *rufipogon* accessions which might be the ancestor of japonica were not included in the samples.

According to the rice diversity researches, *O*. *sativa* could be divided into five subspecies by SSR and SNPs: indica, aus, aromatic or Group V, temperate japonica and tropical japonica [48], [49]. Generally, it is believed that varieties of aromatic, tropical japonica and aus are rarely cultivated in China. Samples of these groups from South and Southeast Asia had been obtained and compared with the cultivated accessions used in the present study. Structure analysis obviously showed that japonica and indica materials used in our research could be divided into temperate japonica and indica population, respectively (Figure S4). Therefore, all conclusions above about japonica should be applicable to temperate japonica. To clearly detect the domestication of the five groups of *O*. *sativa*, varieties of all five groups and *O*. *rufipogon* coming from Southeast and South Asia should be added to the samples.

*O*. *nivara* has been regarded as another ancestor of *O*. *sativa* by genome sequences analysis of 50 accessions of cultivated and wild rice [50]. The two wild progenitors of cultivated rice had genetic divergence and ecological distinction [41]. *O*. *rufipogon* is perennial, photoperiod sensitive and largely cross-fertilized; whereas *O*. *nivara* is annual, photoperiod insensitive and predominantly self-fertilized. *O*. *rufipogon* existed from South China to North Australia, while *O*. *nivara* is mainly found in South and Southeast Asia and have not found in China. Thus, *O*. *nivara* was not included in the present study. To reveal the dynamic process of rice domestication clearly, *O*. *nivara* should be included in the samples in future studies.

## Supporting Information

Figure S1 **Geographic origins of the materials in China.** Red circles indicate indica; blue circles indicate japonica; green triangles indicate *O*. *rufipogon*.
(TIF)

Figure S2 **K value of the Structure analysis (K = 2–10).**
(TIF)

Figure S3 **Haplotype numbers of** ***O*** **.** ***rufipogon*** **from different provinces.**
(TIF)

Figure S4 **Structure of cultivated accessions and varieties of aromatic, tropical japonica and aus.** K = 5. Detailed information about the varieties of aromatic, tropical japonica and aus are shown in Table S3.
(TIF)

Table S1 Collection details of accessions of *O*. *sativa* and *O*. *rufipogon* from China.
(DOC)

Table S2 Summary of the genes sequenced and the primer sequences used in this study.
(DOC)

Table S3 Detailed information of aromatic, tropical japonica and aus in Figure S4.
(DOC)

## References

[1] Smith BD (2006) Eastern North America as an independent center of plant domestication. Proc Natl Acad Sci U S A 103: 12223–12228. 16894156 10.1073/pnas.0604335103 PMC1567861

[2] Jiang L, Liu L (2006) New evidence for the origins of sedentism and rice domestication in the Lower Yangzi River, China. Antiquity 80: 355–361.

[3] Vaughan DA, Morishima H, Kadowaki K (2003) Diversity in the *Oryza* genus. Curr Opin Plant Biol 6: 139–146. 12667870 10.1016/s1369-5266(03)00009-8

[4] Ting Y (1957) The origin and evolution of cultivated rice in China. Acta Agr Sinica 8: 243–260.

[5] Liu Z (1975) The origin and development of cultivated rice in China. Acta Genet Sinica 2: 23–29.

[6] Zong Y, Chen Z, Innes JB, Chen C, Wang Z, et al (2007) Fire and flood management of coastal swamp enabled first rice paddy cultivation in east China. Nature 449: 459–462. 17898767 10.1038/nature06135

[7] Fuller DQ, Qin L, Zheng Y, Zhao Z, Chen X, et al (2009) The domestication process and domestication rate in rice: spikelet bases from the Lower Yangtze. Science 323: 1607–1610. 19299619 10.1126/science.1166605

[8] Gao LZ, Innan H (2008) Nonindependent domestication of the two rice subspecies, *Oryza sativa* ssp. *Indica* and ssp. *japonica*, demonstrated by multilocus microsatellites. Genetics 179: 965–976. 18505887 10.1534/genetics.106.068072 PMC2429889

[9] Molina J, Sikora M, Garud N, Flowers JM, Rubinstein S, et al (2011) Molecular evidence for a single evolutionary origin of domesticated rice. Proc Natl Acad Sci U S A 108: 8351–8356. 21536870 10.1073/pnas.1104686108 PMC3101000

[10] Huang P, Molina J, Flowers JM, Rubinstein S, Jackson SA, et al (2012) Phylogeography of Asian wild rice, *Oryza rufipogon*: a genome-wide view. Mol Ecol 21: 4593–4604. 22646149 10.1111/j.1365-294X.2012.05625.x

[11] Cheng C, Motohashi R, Tsuchimoto S, Fukuta Y, Ohtsubo H, et al (2003) Polyphyletic origin of cultivated rice: based on the interspersion pattern of SINEs. Mol Biol Evol 20: 67–75. 12519908 10.1093/molbev/msg004

[12] Londo JP, Chiang YC, Hung KH, Chiang TY, Schaal BA (2006) Phylogeography of Asian wild rice, *Oryza rufipogon*, reveals multiple independent domestications of cultivated rice, *O*. *sativa* . Proc Natl Acad Sci U S A 103: 9578–9583. 16766658 10.1073/pnas.0603152103 PMC1480449

[13] Huang CL, Hung CY, Chiang YC, Hwang CC, Hsu TW, et al (2012) Footprints of Natural and artificial selection for photoperiod pathway genes in *Oryza* . Plant J 70: 769–782. 22268451 10.1111/j.1365-313X.2012.04915.x

[14] Kumagai M, Wang L, Ueda S (2010) Genetic diversity and evolutionary relationships in genus *Oryza* revealed by using highly variable regions of chloroplast DNA. Gene 462: 44–51. 20450965 10.1016/j.gene.2010.04.013

[15] Dong WP, Liu J, Yu J, Wang L, Zhou SL (2012) Highly variable chloroplast markers for evaluating plant phylogeny at low taxonomic levels and for DNA barcoding. PLoS One 7: e35071. 22511980 10.1371/journal.pone.0035071 PMC3325284

[16] Zhang HL, Zhang DL, Wang MX, Sun JL, Qi YW, et al (2011) A core collection and mini core collection of *Oryza sativa* L. in China. *Theor Appl Genet* 122: 49–61. 20717799 10.1007/s00122-010-1421-7

[17] Lu BR, Cai XX, Jin X (2009) Efficient indica and japonica rice identification based on the InDel molecular method: Its implication in rice breeding and evolutionary research. Prog Nat Sci 19: 1241–1252.

[18] Triboush SO, Danilenko NG, Davydenko OG (1998) A method for isolation of chloroplast DNA and mitochondrial DNA from sunflower. Plant Mol Biol Rep 16: 183–189.

[19] Thompson JD, Gibson Plewniak F, Jeanmougin F, Higgins DG (1997) The CLUSTAL_X windows interface: flexible strategies for multiple sequence alignment aided by quality analysis tools. Nucleic Acids Res 24: 4876–4882. 10.1093/nar/25.24.4876 PMC147148 9396791

[20] Hall TA (1999) Bioedit: A user-friendly biological sequence alignment editor and analysis program for Windows 95/98/NT. Nucleic Acids Symposium Series 41: 95–98.

[21] Nei M (1987) Molecular evolutionary genetics. New York: Columbia University Press.

[22] Watterson GA (1975) On the number of segregating sites in genetical models without recombination. Theor Popul Biol 7: 256–276. 1145509 10.1016/0040-5809(75)90020-9

[23] Rozas J (2009) DNA sequence polymorphism analysis using DnaSP. Methods Mol Biol 537: 337–350. 19378153 10.1007/978-1-59745-251-9_17

[24] Excoffier L, Smouse PE, Quattro JM (1992) Analysis of molecular variance inferred from metric distances among DNA haplotypes: application to human mitochondrial DNA restriction data. Genetics 131: 471–491. 1644282 10.1093/genetics/131.2.479 PMC1205020

[25] Bandelt HJ, Forster P, Rohl A (1999) Median-joining networks for interring intraspecific phylogenies. Mol Biol Evol 16: 37–48. 10331250 10.1093/oxfordjournals.molbev.a026036

[26] Saitou N, Nei M (1987) The neighbor-joining method: a new method for reconstructing phylogenetic trees. Mol Biol Evol 4: 406–425. 3447015 10.1093/oxfordjournals.molbev.a040454

[27] Swofford DL (2002) PAUP*: Phylogenetic Analysis Using Parsimony (and Other Methods), Version 4.0. Sunderland, Massachusetts: Sinauer Associates.

[28] Kimura M (1980) A simple method for estimating evolutionary rates of base substitutions through comparative studies of DNA sequences. J Mol Evol 16: 111–120. 7463489 10.1007/BF01731581

[29] Falush D, Stephens M, Pritchard JK (2003) Inference of population structure: extensions to linked loci and correlated allele frequencies. Genetics 164: 1567–1587. 12930761 10.1093/genetics/164.4.1567 PMC1462648

[30] Wolfe KH, Li WH, Sharp PM (1987) Rates of nucleotide substitution vary greatly among plant mitochondrial, chloroplast, and nuclear DNAs. Proc Natl Acad Sci U S A 84: 9054–9058. 3480529 10.1073/pnas.84.24.9054 PMC299690

[31] Gaut BS, Morton BR, McCaig BC, Clegg MT (1996) Substitution rate comparisons between grasses and palms: synonymous rate differences at the nuclear gene *Adh* parallel rate differences at the plastid gene *rbcL* . Proc Natl Acad Sci U S A 93: 10274–10279. 8816790 10.1073/pnas.93.19.10274 PMC38374

[32] Zheng XM, Ge S (2010) Ecological divergence in the presence of gene flow in two closely related *Oryza* species (*O*. *rufipogon* and *O*. *nivara*). Mol Ecol 19: 2439–2454. 20653085 10.1111/j.1365-294x.2010.04674.x

[33] Sun CQ, Wang XK, Li ZC, Yoshimura A, Iwata N (2001) Comparison of the genetic diversity of common wild rice (*Oryza rufipogon* Griff.) and cultivated rice (*Oryza sativa* L.) using RFLP markers. Theor Appl Genet 102: 157–162.

[34] Gao LZ (2004) Population structure and conservation genetics of wild rice *Oryza rufipogon* (Poaceae): a region-wide perspective from microsatellite variation. Mol Ecol 13: 1009–1024. 15078440 10.1111/j.1365-294X.2004.02108.x

[35] Wang MX, Zhang HL, Zhang DL, Qi YW, Fan ZL, et al (2008) Genetic structure of *Oryza rufipogon* Griff. in China. Heredity 101: 527–535. 18827837 10.1038/hdy.2008.61

[36] Zhu QH, Ge S (2005) Phylogenetic relationships among A-genome species of the genus *Oryza* revealed by intron sequences of four nuclear genes. New Phytol 167: 249–265. 15948847 10.1111/j.1469-8137.2005.01406.x

[37] Zhu Q, Zheng X, Luo J, Gaut BS, Ge S (2007) Multilocus analysis of nucleotide variation of *Oryza sativa* and its wild relatives: severe bottleneck during domestication of rice. Mol Biol Evol 24: 875–888. 17218640 10.1093/molbev/msm005

[38] Rakshit S, Rakshit A, Matsumura H, Takahashi Y, Hasegawa Y, et al (2007) Large-scale DNA polymorphism study of *Oryza sativa* and *O*. *rufipogon* reveals the origin and divergence of Asian rice. Theor Appl Genet 114: 731–743. 17219210 10.1007/s00122-006-0473-1

[39] He Z, Zhai W, Wen H, Tang T, Wang Y, et al (2011) Two evolutionary histories in the genome of rice: the roles of domestication genes. PLoS Genet 7: e1002100. 21695282 10.1371/journal.pgen.1002100 PMC3111475

[40] Yang C, Kawahara Y, Mizuno H, Wu J, Matsumoto T, et al (2011) Independent domestication of Asian rice followed by gene flow from japonica to indica. Mol Bio Evol 29: 1471–1479. 22319137 10.1093/molbev/msr315

[41] Vaughan DA, Lu BR, Tomooka N (2008) The evolving story of rice evolution. Plant Sci 174: 394–408.

[42] Wei X, Qiao WH, Chen YT, Wang RS, Cao LR, et al (2012) Domestication and geographic origin of *Oryza sativa* in China: insights from multilocus analysis of nucleotide variation of *O*. *sativa* and *O*. *rufipogon* . Mol Ecol 21: 5073–5087. 22989375 10.1111/j.1365-294X.2012.05748.x

[43] Sweeney M, McCouch S (2007) The complex history of the domestication of rice. Ann Bot 100: 951–957. 17617555 10.1093/aob/mcm128 PMC2759204

[44] Kovach MJ, Sweeney MT, McCouch SR (2007) New insights into the history of rice domestication. Trends Genet 23: 578–587. 17963977 10.1016/j.tig.2007.08.012

[45] Sang T, Ge S (2007a) Genetics and phylogenetics of rice domestication. Curr Opin Genet and Dev 17: 533–538. 17988855 10.1016/j.gde.2007.09.005

[46] Sang T, Ge S (2007b) The puzzle of rice domestication. J Integr Plant Biol 49: 760–768.

[47] Tang J, Xia H, Cao M, Zhang X, Zeng W, et al (2004) A comparison of rice chloroplast genomes. Plant physiol 135: 412–420. 15122023 10.1104/pp.103.031245 PMC429394

[48] Garris AJ, Tai TH, Coburn J, Kresovich S, McCouch S (2005) Genetic structure and diversity in *Oryza sativa* L. Genetics. 169: 1631–1638. 10.1534/genetics.104.035642 PMC1449546 15654106

[49] Zhao K, Wright M, Kimball J, Eizenga G, McClung A, et al (2010) Genomic diversity and introgression in *O*. *sativa* reveal the impact of domestication and breeding on the rice genome. PLoS One 5: e10780. 20520727 10.1371/journal.pone.0010780 PMC2875394

[50] Xu X, Liu X, Ge S, Jensen DJ, Hu F, et al (2012) Resequencing 50 accessions of cultivated and wild rice yields markers for identifying agronormically important genes. Nat Biotechnol 30: 105–111. 10.1038/nbt.2050 22158310

