# Supplementary figures and images for: Origin of Oryza sativa in China Inferred by Nucleotide Polymorphisms of Organelle DNA
Source: PLoS One. 2012 Nov 15;7(11):e49546. doi: 10.1371/journal.pone.0049546 (PMC3499492; doi:10.1371/journal.pone.0049546)

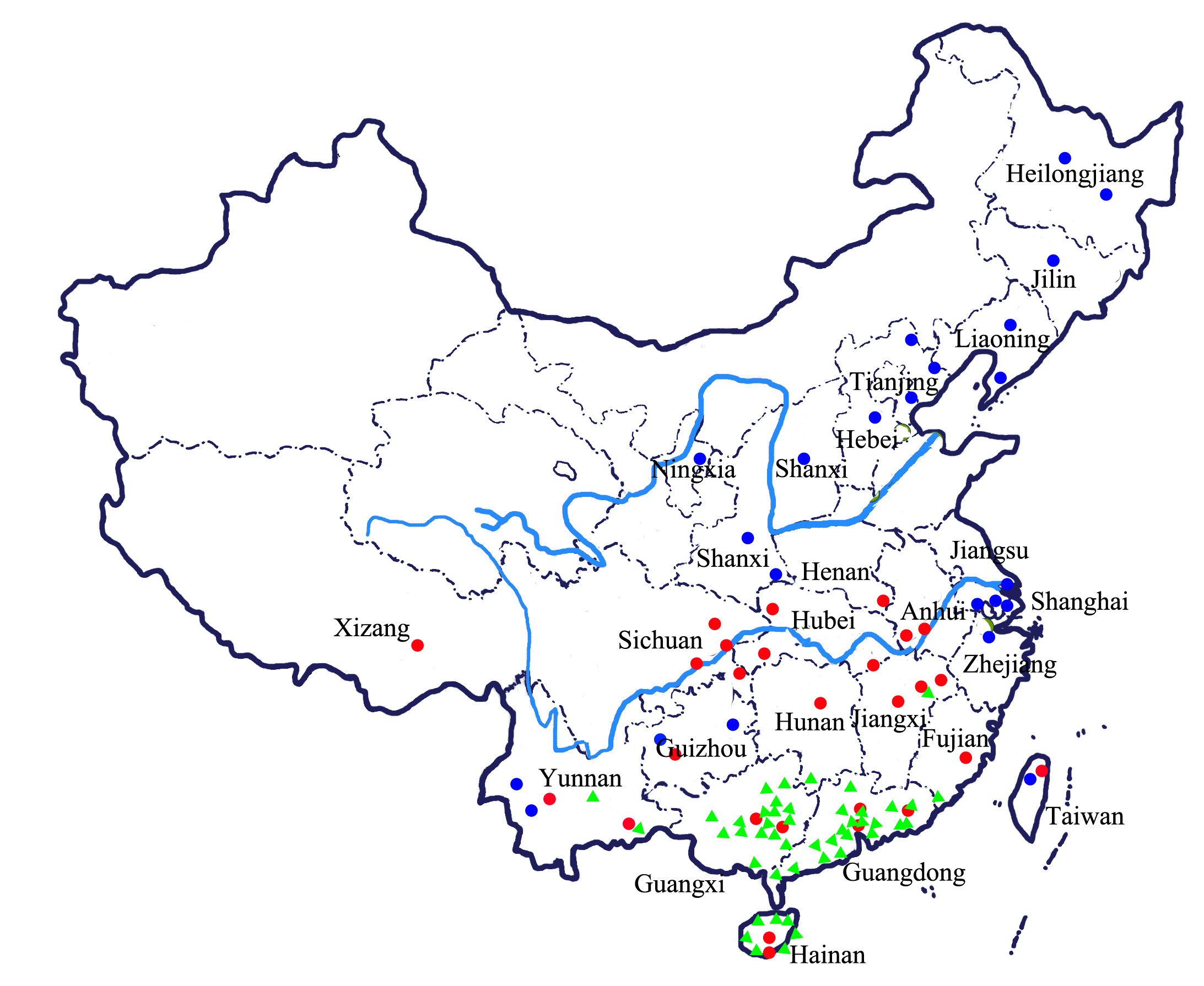

Supplement: Figure S1 — Geographic origins of the materials in China. Red circles indicate indica; blue circles indicate japonica; green triangles indicate O. rufipogon. (TIF) [file pone.0049546.s001.tif]

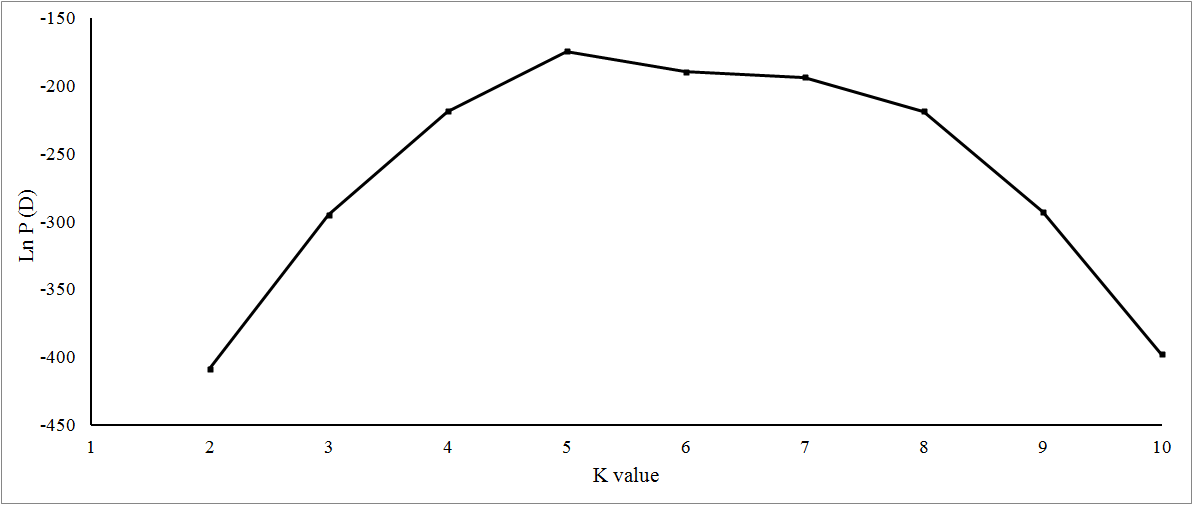

Supplement: Figure S2 — K value of the Structure analysis (K = 2–10). (TIF) [file pone.0049546.s002.tif]

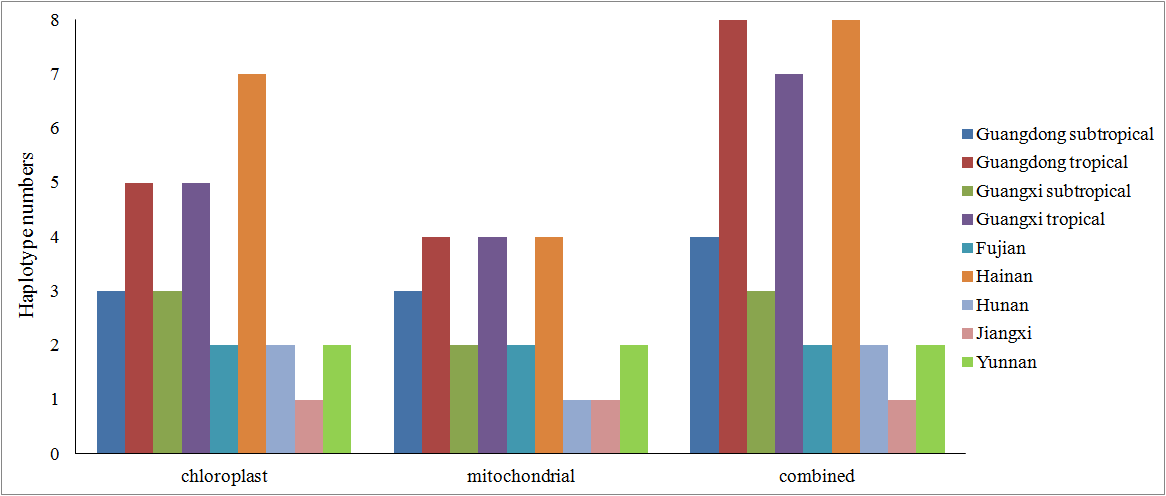

Supplement: Figure S3 — Haplotype numbers of O . rufipogon from different provinces. (TIF) [file pone.0049546.s003.tif]

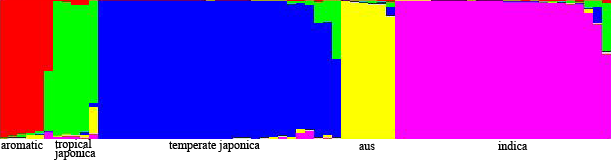

Supplement: Figure S4 — Structure of cultivated accessions and varieties of aromatic, tropical japonica and aus. K = 5. Detailed information about the varieties of aromatic, tropical japonica and aus are shown in Table S3. (TIF) [file pone.0049546.s004.tif]
