# Supplementary material for: Origin of Oryza sativa in China Inferred by Nucleotide Polymorphisms of Organelle DNA
Source: PLoS One. 2012 Nov 15;7(11):e49546. doi: 10.1371/journal.pone.0049546 (PMC3499492; doi:10.1371/journal.pone.0049546)
Supplement: Table S1 — Collection details of accessions of O. sativa and O. rufipogon from China. (DOC) [file pone.0049546.s005.doc]

**Table S1. Collection details of accessions of *O. sativa* and *O. rufipogon* from China.**

| **Taxa** | **Samples Location** | **Code** | **n** | **Latitude (N)** | **Longitude (E)** | **Haplotype** | **Cluster** |
| --- | --- | --- | --- | --- | --- | --- | --- |
| japonica | Guizhou, Qinglong | GZ_QL-J | 1 | 105°12′ | 25°50′ | 1 | A |
|  | Guizhou, Shibing | GZ_SB-J | 1 | 108°08′ | 27°03′ | 1 | A |
|  | Hebei, Funing | HeB_FN-J | 1 | 119°14′ | 39°53′ | 1 | A |
|  | Hebei, Longhui | HeB_LH-J | 1 | 117°43′ | 41°18′ | 1 | A |
|  | Hebei, Gaoyang | HeB_GY-J | 1 | 115°46′ | 38°41′ | 1 | A |
|  | Heilongjiang, Hailin | HLJ_HL-J | 1 | 129°23′ | 44°34′ | 1 | A |
|  | Heilongjiang, Suihua | HLJ_SH-J | 1 | 126°59′ | 46°38′ | 1 | A |
|  | Jilin, Huaide | JL_HD-J | 1 | 124°48′ | 43°31′ | 1 | A |
|  | Jiangsu, Jiangyin | JS_JY-J | 1 | 120°17′ | 31°40′ | 1 | A |
|  | Jiangsu, Nantong | JS_NT-J | 1 | 121°05′ | 32°06′ | 1 | A |
|  | Jiangsu, Wujin | JS_WJ-J | 1 | 119°58′ | 31°47′ | 1 | A |
|  | Liaoning, Dandong | LN_DD-J | 1 | 124°20′ | 40°03′ | 1 | A |
|  | Liaoning, Gaixian | LN_GX-J | 1 | 122°21′ | 40°25′ | 1 | A |
|  | Ningxia, Zhongwei | NX_ZW-J | 1 | 105°11′ | 37°31′ | 1 | A |
|  | Shanxi, Lantian | SaX_LT-J | 1 | 109°05′ | 34°32′ | 1 | A |
|  | Shanxi, Shanyang | SaX_SY-J | 1 | 109°52′ | 33°31′ | 1 | A |
|  | Shanghai, Fengxian | SHFX-J | 1 | 121°27′ | 30°55′ | 1 | A |
|  | Shanxi, Taiyuan | SX-TY-J | 1 | 112°03′ | 37°13′ | 1 | A |
|  | Tianjing, Tianjing | TJ-TJ-J | 1 | 117°39′ | 39°01′ | 1 | A |
|  | Taiwan, Taibei | TW-TB-J | 1 | 121°31′ | 25°03′ | 1 | A |
|  | Yunnan, Longling | YN_LL-J | 1 | 98°41′ | 24°36′ | 1 | A |
|  | Yunnan, Malong | YN_ML-J | 1 | 103°33′ | 25°25′ | 1 | A |
|  | Zhejiang, Wuxing | ZJ_WX-J | 1 | 120°06′ | 30°52′ | 1 | A |
| indica | Anhui, Huaining | AH_HN-I | 1 | 116°39′ | 30°24′ | 2 | B |
|  | Anhui, Taihu | AH_TH-I | 1 | 116°17′ | 30°26′ | 2 | B |
|  | Fujian, Xianyou | FJ_XY-I | 1 | 118°42′ | 25°27′ | 2 | B |
|  | Guangdong, Guangzhou | GD-GZ-I | 1 | 113°17′ | 23°08′ | 2 | B |
|  | Guangdong, Longchuan | GD_LC-I | 1 | 115°15′ | 24°06′ | 2 | B |
|  | Guangdong, Yingde | GD_YD-I | 1 | 113°24′ | 24°10′ | 3 | C |
|  | Guizhou, Wuchuan | GZ_WC-I | 1 | 107°53′ | 28°31′ | 2 | B |
|  | Guangxi, Fengshan | GX_FeS-I | 1 | 107°03′ | 24°31′ | 3 | C |
|  | Guangxi, Hengxian | GX_HX-I | 1 | 109°16′ | 22°41′ | 2 | B |
|  | Hainan, Baoting | HN_BT-I | 1 | 109°41′ | 18°38′ | 2 | B |
|  | Hainan, Sanya | HN_SY-I | 1 | 109°31′ | 18°15′ | 2 | B |
|  | Henan, Huaibin | HeN_HB-I | 1 | 115°25′ | 32°27′ | 2 | B |
|  | Hubei, Puqi | HuB_PQ-I | 1 | 113°53′ | 29°43′ | 2 | B |
|  | Hubei, Xuanen | HuB_XE-I | 1 | 109°23′ | 29°59′ | 2 | B |
|  | Hubei, Zhuxi | HuB_ZX-I | 1 | 109°41′ | 32°19′ | 2 | B |
|  | Hunan, Changsha | HuN-1-I | 1 | 112°58′ | 28°11′ | 2 | B |
|  | Jiangxi, Dongxiang | JX_DX-I | 1 | 116°31′ | 28°16′ | 2 | B |
|  | Jiangxi, Guixi | JX_GX-I | 1 | 117°13′ | 28°18′ | 2 | B |
|  | Jiangxi, Nanchang | JX-NC-I | 1 | 115°56′ | 28°32′ | 2 | B |
|  | Sichuan, Gulan | SC_GL-I | 1 | 105°48′ | 28°02′ | 2 | B |
|  | Sichuan, Quxian | SC_QX-I | 1 | 106°58′ | 30°15′ | 2 | B |
|  | Sichuan, Wanxian | SC_WX-I | 1 | 108°21′ | 30°50′ | 2 | B |
|  | Sichuan, Yibin | SC_YB-I | 1 | 104°37′ | 28°46′ | 2 | B |
|  | Taiwan, Taibei | TW-TB-I | 1 | 121°31′ | 25°03′ | 2 | B |
|  | Xizang, Motuo | XZ_MT-I | 1 | 95°19′ | 29°18′ | 2 | B |
|  | Yunnan, Jinping | YN_JP-I | 1 | 103°14 | ′22°47′ | 2 | B |
|  | Yunnan, Lincang | YN_LiC-I | 1 | 100°05′ | 23°53′ | 2 | B |
| subtropical *O. rufipogon* | Fujiang, Zhangpu | FJ_ZP-W | 8 | 117°36′ | 24°10′ | 1,5 | A,D |
|  | Guangdong, Conghua | GD_CH-W | 1 | 113°34′ | 23°33′ | 9 | E |
|  | Guangdong, Fogang | GD_FG-W | 2 | 113°31′ | 23°52′ | 1 | A |
|  | Guangdong, Heyuan | GD_HeY-W | 2 | 114°41′ | 23°44′ | 9 | E |
|  | Guangdong, Huaxian | GD_HX-W | 2 | 113°12′ | 23°40′ | 1,4 | A,D |
|  | Guangdong, Qujiang | GD_QJ-W | 2 | 113°36′ | 24°41′ | 1 | A |
|  | Guangdong, Qingyuan | GD_QY-W | 1 | 113°01′ | 23°42′ | 1 | A |
|  | Guangdong, Renhua | GD_RH-W | 1 | 113°44′ | 25°06′ | 1 | A |
|  | Guangdong, Zijin | GD_ZJ-W | 3 | 115°11′ | 23°39′ | 1,9,10 | A,D,E |
|  | Guangxi, Guilin | GX_GL-W | 1 | 110°15′ | 25°11′ | 1 | A |
|  | Guangxi, Guiping | GX_GP-W | 2 | 110°05′ | 23°27′ | 1,5 | A,D |
|  | Guangxi, Laibin | GX_LB-W | 2 | 109°14′ | 23°44′ | 1,9 | A,E |
|  | Guangxi, Luzhai | GX_LZ-W | 1 | 109°44′ | 24°29′ | 1 | A |
|  | Guangxi, Pingnan | GX_PN-W | 2 | 110°23′ | 23°33′ | 1,5 | A,D |
|  | Guangxi, Wuxuan | GX_WX-W | 2 | 109°40′ | 23°36′ | 1 | A |
|  | Guangxi, Yongfu | GX_YF-W | 1 | 110°00′ | 24°59′ | 1 | A |
|  | Hunan, Chaling | HuN_CL-W | 3 | 113°33′ | 26°48′ | 1,15 | A |
|  | Hunan, Jiangyong | HuN_JY-W | 3 | 111°20′ | 25°07′ | 1 | A |
|  | Jiangxi, Dongxiang | JX_DX-W | 3 | 116°31′ | 28°16′ | 5 | D |
|  | Yunnan, Yuanjiang | YN_YJ-W | 4 | 102°00′ | 23°59′ | 4 | D |
| tropical *O. rufipogon* | Guangdong, Deqing | GD_DQ-W | 1 | 111°46′ | 23°09′ | 1 | A |
|  | Guangdong, Enping | GD_EP-W | 3 | 112°19′ | 22°12′ | 1,3,4 | A,C,D |
|  | Guangdong, Gaoming | GD_GM-W | 1 | 112°53′ | 22°54′ | 1 | A |
|  | Guangdong, Huidong | GD_HD-W | 2 | 114°43′ | 22°59′ | 1 | A |
|  | Guangdong, Haifeng | GD_HF-W | 2 | 115°15′ | 22°58′ | 1,4 | A,D |
|  | Guangdong, Huiyang | GD_HY-W | 2 | 114°40′ | 23°09′ | 1,9 | A,E |
|  | Guangdong, Huizhou | GD_HZ-W | 1 | 114°24′ | 23°03′ | 1 | A |
|  | Guangdong, Kaiping | GD_KP-W | 2 | 112°41′ | 22°22′ | 1,5 | A,D |
|  | Guangdong, Miaoming | GD_MM-W | 1 | 110°53′ | 21°40′ | 3 | C |
|  | Guangdong, Puning | GD_PN-W | 1 | 116°10′ | 23°14′ | 3 | C |
|  | Guangdong, Suixi | GD_SX-W | 2 | 110°15′ | 21°23′ | 8,13 | A,D |
|  | Guangdong, Taishan | GD_TS-W | 2 | 112°47′ | 22°15′ | 1,3 | A,C |
|  | Guangdong, Yangchun | GD_YC-W | 1 | 111°43′ | 22°18′ | 10 | D |
|  | Guangxi, binyang | GX_BY-W | 1 | 108°48′ | 23°13′ | 1 | A |
|  | Guangxi, Fusui | GX_FS-W | 2 | 107°31′ | 22°11′ | 5,10 | D |
|  | Guangxi, Guigang | GX_GG-W | 2 | 109°36′ | 23°06′ | 1 | A |
|  | Guangxi, Guixian | GX_GX-W | 2 | 109°36′ | 23°10′ | 1 | A |
|  | Guangxi, Hepu | GX_HP-W | 1 | 109°12′ | 21°40′ | 2 | B |
|  | Guangxi, Hengxian | GX_HX-W | 1 | 109°16′ | 22°41′ | 1 | A |
|  | Guangxi, Longan | GX_LA-W | 5 | 107°41′ | 23°10′ | 1,4 | A,D |
|  | Guangxi, Rongxian | GX_RX-W | 1 | 110°33′ | 22°51′ | 1 | B |
|  | Guangxi, Hepu | GX_HP-W | 2 | 109°12′ | 21°40′ | 3,4 | C,D |
|  | Guangxi, Wuming | GX_WM-W | 1 | 108°16′ | 23°09′ | 1 | A |
|  | Guangxi, Yulin | GX_YL-W | 3 | 110°08′ | 22°38′ | 1,9 | A,E |
|  | Guangxi, Yongning | GX_YN-W | 3 | 108°29′ | 22°46′ | 5,14 | D |
|  | Hainan, Changjiang | HN_CJ-W | 1 | 109°03′ | 19°16′ | 2 | B |
|  | Hainan, Dingan | HN_DA-W | 2 | 110°20′ | 19°42′ | 4,12 | B |
|  | Hainan, Haikou | HN_HK-W | 3 | 110°20′ | 20°03′ | 3,7 | C |
|  | Hainan, Ledong | HN_LD-W | 3 | 109°10′ | 18°45′ | 2,4 | B |
|  | Hainan, Lingao | HN_LG-W | 6 | 109°41′ | 19°54′ | 3,5,6 | A,C,D |
|  | Hainan, Qionghai | HN_QH-W | 1 | 110°27′ | 19°14′ | 2 | B |
|  | Hainan, Qiongshan | HN_QS-W | 5 | 110°33′ | 19°57′ | 3,5 | C,D |
|  | Hainan, Sanya | HN_SY-W | 2 | 109°31′ | 18°15′ | 2,11 | A,B |
|  | Yunnan, Jinghong | YN_JH-W | 6 | 100°48′ | 22°01′ | 9 | E |

n represents the number of the accessions for the landraces and *O*. *rufipogon*.
