# Supplementary material for: Origin of Oryza sativa in China Inferred by Nucleotide Polymorphisms of Organelle DNA
Source: PLoS One. 2012 Nov 15;7(11):e49546. doi: 10.1371/journal.pone.0049546 (PMC3499492; doi:10.1371/journal.pone.0049546)
Supplement: Table S2 — Summary of the genes sequenced and the primer sequences used in this study. (DOC) [file pone.0049546.s006.doc]

**Table S2.** Summary of the genes sequenced and the primer sequences used in this study.

| **Loci** | **Organelle** | **Primer** | **Sequence (5’-3’)** |
| --- | --- | --- | --- |
| *rps16* | chloroplast | rps16-F | GCTCTTTGCGGAGTCTTT |
|  |  | rps16-R | ATGTTGGATTGGCACGAC |
| *trnG-trnfM* | chloroplast | trnG-trnfM-F | GCCGTTTATTCGGATTGT |
|  |  | trnG-trnfM-R | AGCGGAGTAGAGCAGTTT |
| *atpA* | chloroplast | atpA-1-F | GAATCAGGTCCGACAACG |
|  |  | atpA-1-R | ACGGAGGATGCTCTTTGA |
|  |  | atpA-2-F | CTTACTTGGGTCGTGTTA |
|  |  | atpA-2-R | GGACGCAATCTTATTTCTAT |
| *trnT-trnL* | chloroplast | trnT-trnL-1-F | CTTCCGGATTTAGGTGT |
|  |  | trnT-trnL-1-R | AAATAGATTTGCGAATTAGAG |
|  |  | trnT-trnL-2-F | AATGCGATGCTCTAACCT |
|  |  | trnT-trnL-2-R | AGCCCTTCTTTCCCTAAT |
| *trnC-ycf6* | chloroplast | trnC-ycf6-F | ATAAAGTTCGGCAAAATGAT |
|  |  | trnC-ycf6-R | AAGGGAAGTTTTCTAGTGTTAG |
| *cox3* | mitochondrial | cox3-F | TATGAAATATCTCAAACCCACG |
|  |  | cox3-R | GGGCATGATAAAGACCAATAA |
| *cox1* | mitochondrial | cox1-1-F | ATGACGGAGCCTAGACCTT |
|  |  | cox1-1-R | AATGACAAATCTGGTCCGAT |
|  |  | cox1-2-F | GAGCCTAGACCTTTTCTT |
|  |  | cox1-2-R | ATTAACTCTGCGATTTTG |
| *nhd4* | mitochondrial | nhd4-F | CAAAGTACACCTCTTCCGTAG |
|  |  | nhd4-R | AAGTCGCTTACTCTCCGTCTA |
| *rps2-trnfM* | mitochondrial | rps2-trnfM-F | ATTGCTTTGTTAGAGGGAG |
|  |  | rps2-trnfM-R | TATTCGCAGGTAAATTCGG |
