# Supplementary material for: Origin of Oryza sativa in China Inferred by Nucleotide Polymorphisms of Organelle DNA
Source: PLoS One. 2012 Nov 15;7(11):e49546. doi: 10.1371/journal.pone.0049546 (PMC3499492; doi:10.1371/journal.pone.0049546)
Supplement: Table S3 — Detailed information of aromatic, tropical japonica and aus in Figure S4. (DOC) [file pone.0049546.s007.doc]

Table S3 Detailed information about the varieties of aromatic, tropical japonica and aus used in Figure S4.

| Group | IRGC NO. | Name | Origin |
| --- | --- | --- | --- |
| aus | 6264 | N 22 | India |
|  | 6307 | JHONA 349 | India |
|  | 8839 | DV 85 | Bangladesh |
|  | 12440 | ARC 10352 | India |
|  | 25901 | MIRITI | Bangladesh |
|  | 45195 | BJ 1 | India |
| tropical japonica | 3575 | TONDOK | Indonesia |
|  | 3782 | KINASTANO | Philippines |
|  | 8261 | PADI KASALLE | Indonesia |
|  | 16428 | GUNDIL KUNING | Indonesia |
|  | 43394 | GOGO LEMPUK | Indonesia |
|  | 117710 | DHALA SHALTTA | India |
| aromatic | 5857 | DA 13 | Bangladesh |
|  | 5999 | PANKHARI 203 | India |
|  | 9060 | JC 101 | India |
|  | 9062 | JC 111 | India |
|  | 42469 | ARC 13829 | India |
|  | 53637 | BASMATI 217 | India |
